# Kelley’s Paradox and strength skewness in research on unconscious mental processes

**DOI:** 10.3758/s13423-024-02578-1

**Published:** 2024-10-15

**Authors:** Daryl Y. H. Lee, Christopher J. Berry, David R. Shanks

**Affiliations:** 1 Department of Experimental Psychology, University College London, 26 Bedford Way, London, WC1H 0AP UK; 2 School of Psychology, University of Plymouth, Plymouth, UK

**Keywords:** Implicit memory, Models of recognition memory, Recognition memory, Word recognition

## Abstract

A widely adopted approach in research on unconscious perception and cognition involves contrasting behavioral or neural responses to stimuli that have been presented to participants (e.g., old items in a memory test) against those that have not (e.g., new items), and which participants do not discriminate in their conscious reports. We demonstrate that such contrasts do not license inferences about unconscious processing, for two reasons. One is Kelley’s Paradox, a statistical phenomenon caused by regression to the mean. In the inevitable presence of measurement error, true awareness of the contrasted stimuli is not equal. The second is a consequence, within the framework of Signal Detection Theory, of unequal skewness in the strengths of target and nontarget items. The fallacious reasoning that underlies the employment of this contrast methodology is illustrated through both computational simulations and formal analysis, and its prevalence is documented in a narrative literature review. Additionally, a recognition memory experiment is reported which tests and confirms a prediction of our analysis of the contrast methodology and corroborates the susceptibility of this method to artifacts attributable to Kelley’s Paradox and strength skewness. This work challenges the validity of conclusions drawn from this popular analytic approach.

## Introduction

In experimental psychology, post hoc data selection is a longstanding practice, with examples spanning back to seminal works (e.g., Lazarus & McCleary, 1951; Peirce & Jastrow, 1884; Sidis, 1898; Williams, 1938). At its core, this method is predicated on the selection of either specific participants or trials for subsequent analysis on a measure, contingent upon their responses on another measure (for an in-depth review, see Shanks, 2017). This method is especially popular in studies of unconscious cognitive mechanisms, including unconscious memory and perception.

Broadly, post hoc data selection leads to two divergent approaches. The first approach, which may be termed *post hoc subgroup selection*, has been widely applied in subliminal perception and unconscious learning studies (e.g., Chien et al., 2022; Sklar et al., 2012; Stein et al., 2020; Zhang & Carlisle, 2023). This approach hinges on the selection of participants or trials where awareness of stimuli is absent (e.g., Sheikh et al., 2019), while those showing evidence of awareness are removed from the analysis. Such a selection aims to ensure that any observed behavioral or neural effects can be attributed to unconscious processes. Despite its prevalence, the inherent shortcomings of this method have been much discussed over the past several years (Rothkirch et al., 2022; Shanks, 2017; Shanks et al., 2021; Yaron et al., 2023). Notably, Shanks (2017) illuminated the inherent pitfalls of post hoc selection when investigating unconscious processing. By only including participants with low awareness scores, there is a risk of bias in assessing their true awareness levels. The apparent evidence of unconscious processing in a post hoc selected group could stem from the inclusion of participants who, despite being conscious of the stimuli, were incorrectly classified as unaware.

In the current article, our main focus is on a second, related but conceptually distinct, analytic approach. Here, attention is turned to contrasts between pairs of (sets of) stimuli or trials. For example, within a two-stage recognition memory procedure, commonly adopted in memory research (e.g., Kark et al., 2020; Ramey et al., 2019; Rugg et al., 1998), participants are first presented with a set of stimuli one at a time and then later encounter these same stimuli mixed with new ones. In the second, test, stage they judge whether each item was presented in the first stage. Analyses focus on contrasting responses to stimuli that participants fail to recognize from the initial phase (“misses”) with truly new stimuli that are correctly identified (“correct rejections” or CRs). This contrast is then interpreted as ensuring matching of awareness, and hence any behavioral or neural difference between the misses and CRs must be evidence of unconscious processing. In what may be the first use of the method, Rugg et al. (1998), in an article published in *Nature*, asked whether the neural correlates of implicit and explicit memory could be dissociated. Adopting the recognition design described above, they extracted misses and CRs and then measured a neural signal (event-related potentials, ERPs) associated with these stimuli. Since participants made the same recognition response to these, it was inferred that their strengths of conscious mental representation were equal. As articulated by Rugg et al. (1998):

Crucially, we compared the ERPs produced by new words with those produced by old words that were misclassified by the subjects as new, reasoning that differences between these two classes of ERP would reflect memory in the absence of awareness. (p. 595).

In short, the approach involves a two-step process, where stimuli classified as misses and correct rejections are first selected, followed by asking whether the miss-correct rejection contrast identifies any other indirect or implicit outcome of interest.

In the perceptual domain the procedure is conceptually similar to that described above for memory, but in this case a contrast is created between stimulus-present and stimulus-absent events for which participants report stimulus absence (i.e., misses and CRs). For instance, in an investigation on visual perception utilizing the attentional blink paradigm, Marois et al. (2004) compared neural responses during failure in stimulus detection (i.e., misses) against correct reports of stimulus absence (i.e., CRs). A significant difference in neural activation within the parahippocampal place area was revealed, and the authors attributed this result to unconscious perception.

Although the miss-CR contrast approach shares procedural similarities with the post hoc subgroup selection approach, as a subgroup of stimuli is selected based on certain criteria, it differs from the latter in its focus on drawing inferences by contrasting misses and CRs. For the sake of clarity, we hereafter refer to this approach as the *miss-CR contrast* approach. These approaches share the overarching aim of isolating the role of unconscious processing by controlling for or eliminating any influence of awareness (e.g., Ramey et al., 2020; Ramey et al., 2019). It should be noted that the miss-CR contrast qualifies as a type of post hoc data selection: while the experimenter predetermines the old/new status of a test stimulus (i.e., item), its categorization as a miss or CR is determined post hoc based on the participant’s report (Lee & Shanks, 2023).

While Shanks (2017) highlighted the risks of drawing erroneous conclusions based on post hoc data selection, that analysis primarily concentrated on the post hoc subgroup selection approach. The present work aims to scrutinize the limitations of the miss-CR contrast approach, building upon the foundations set by Shanks (2017). As a preview, we first discuss Kelley’s Paradox – a counterintuitive statistical phenomenon – and demonstrate how it arises when the miss-CR contrast approach is applied through simulations. As we delve deeper into this phenomenon, we discuss another fundamental statistical concept – regression to the mean (RttM) – which is closely linked to Kelley’s Paradox. In a formal analysis within the framework of Signal Detection Theory (SDT), we show that the key assumption on which the miss-CR approach rests is usually false. This analysis also reveals a second factor at play, namely a property of intervals under target and nontarget SDT distributions – differences in *strength skewness* – which invalidates a fundamental and indispensable assumption of the miss-CR contrast approach. This will be followed by a narrative literature review illustrating the widespread adoption of this approach. Finally, we report an experiment that directly tests for the involvement of Kelley’s Paradox and strength skewness in research on unconscious memory.

### Kelley’s Paradox

To understand the invalidity of the miss-CR contrast approach, consider the following hypothetical scenario. Imagine that we have data on the medical school application scores of a large group of students who come from relatively disadvantaged backgrounds and another large group who come from relatively advantaged backgrounds, and assume also that the scores for the advantaged group are higher on average than those of the disadvantaged group. The distribution of scores in the advantaged group is shifted upwards compared to that of the disadvantaged group. We select all those students whose scores fall in a narrow interval. Some of these are members of the disadvantaged group and others of the advantaged group, but we have chosen them to be approximately equated in their application scores. Now we fast forward to the exam scores they achieve in their final medical school exams some years later. We confidently predict that the subgroups will be equivalent or that the disadvantaged subgroup will outperform the advantaged subgroup (because their disadvantage held them back and their true potential now has an opportunity to reveal itself). Somewhat to our surprise, we find that the advantaged subgroup outperforms the disadvantaged subgroup. We infer that advantage has persisted through medical training.

What is the problem with this scenario? It is that it rests on a false assumption. We are implicitly assuming that we have created two subgroups matched for their initial ability, since after all we specifically selected them to have equivalent application scores. *But the true scores of the two subgroups are not matched*. Hence whatever outcome we see in the final medical school exam scores will be confounded by differences in application scores. In the presence of any non-zero degree of measurement error (and indeed under some conditions that we explicate below, even in the absence of measurement error), the true score of the advantaged subgroup will be higher than that of the disadvantaged one (see Wainer & Brown, 2006, for an illustration based on real educational attainment data). The key point is that one cannot double-dip by using scores both to create a subgroup and to estimate the mean in that subgroup.

We now formalize and simulate an abstract version of this scenario. Consider two groups differing in a variable, measured with some error. According to classical test theory (Hambleton & Jones, 1993), such errors cancel out in aggregate, leading to an alignment between the true and observed score means for each group. Imagine that we construct two subgroups, one from each group, who score identically (or within an interval, including one bounded by either -∞ or +∞) on the variable. It is natural to assume that the latent scores of these sub-groups are identical. But now we measure them on this variable again. This time they score differently. Why is this? The reason is simply an extension of the regression effect that occurs in post hoc selection (Shanks, 2017). On the second measurement, with independent errors, the scores of members from the subgroup with a higher true mean will regress towards their group mean, while those of members of the subgroup with a lower true mean will regress towards their group mean. Because these two group means differ, then so will the true scores of the two subgroups.

The fact that members from the two subgroups, despite having scored identically (or similarly) on the first measurement, may score differently on the subsequent measurement provides insight into the pitfalls of the miss-CR contrast approach. This methodological problem was coined Kelley’s Paradox by Wainer and Brown (2006), after statistician Truman Kelley who described the underlying statistical phenomenon nearly a century ago (Kelley, 1927; see also Smith, 2017). It is not a logical paradox like the liar paradox (e.g., Greenough, 2001). Instead, it arises from counterintuitive findings that are statistically sound but which challenge established beliefs (Wainer, 2000; Wainer & Brown, 2006). To elucidate Kelley’s Paradox, we present a simple simulation rooted in classical test theory. In this framework, any given observed score equates to the sum of a true score and an error term (Lord & Novick, 1968):

1 $${X}_{i}={T}_{i}+ {E}_{i}$$

where ${X}_{i}$ denotes the observed score, ${T}_{i}$ represents the true score, and ${E}_{i}$ is the error term associated with the measurement, for a given individual $i$. Two groups were constructed for this simulation: the advantaged group and the disadvantaged group. For each individual in these groups, a true score was randomly sampled from a normal distribution, modeled as:

2 $${T}_{i} \sim N({\mu }_{g},{{\sigma }_{T}}^{2})$$

where ${\mu }_{g}$ is the group mean (set at 60 for the advantaged group and at 40 for the disadvantaged group), and ${{\sigma }_{T}}^{2}$ represents the variance for the normal distribution (set at 100 for both groups). While measurements contain inherent errors, in line with classical test theory, this error, having a zero mean, is not correlated with the true scores. Accordingly, an error term for each participant was randomly sampled from a zero-centered normal distribution:

3 $${E}_{i} \sim N(0,{{\sigma }_{E}}^{2})$$

where ${{\sigma }_{E}}^{2}$ represents the variance for the normal distribution (set at 100 for both groups, mirroring the variance of the true scores). To compute the observed scores – the scores we would discern in a real-world setting – we summed the true scores and the error components via Eq. (1). Our simulation comprised 50,000 individuals for each group, totaling 100,000 participants.

**Fig. 1:**
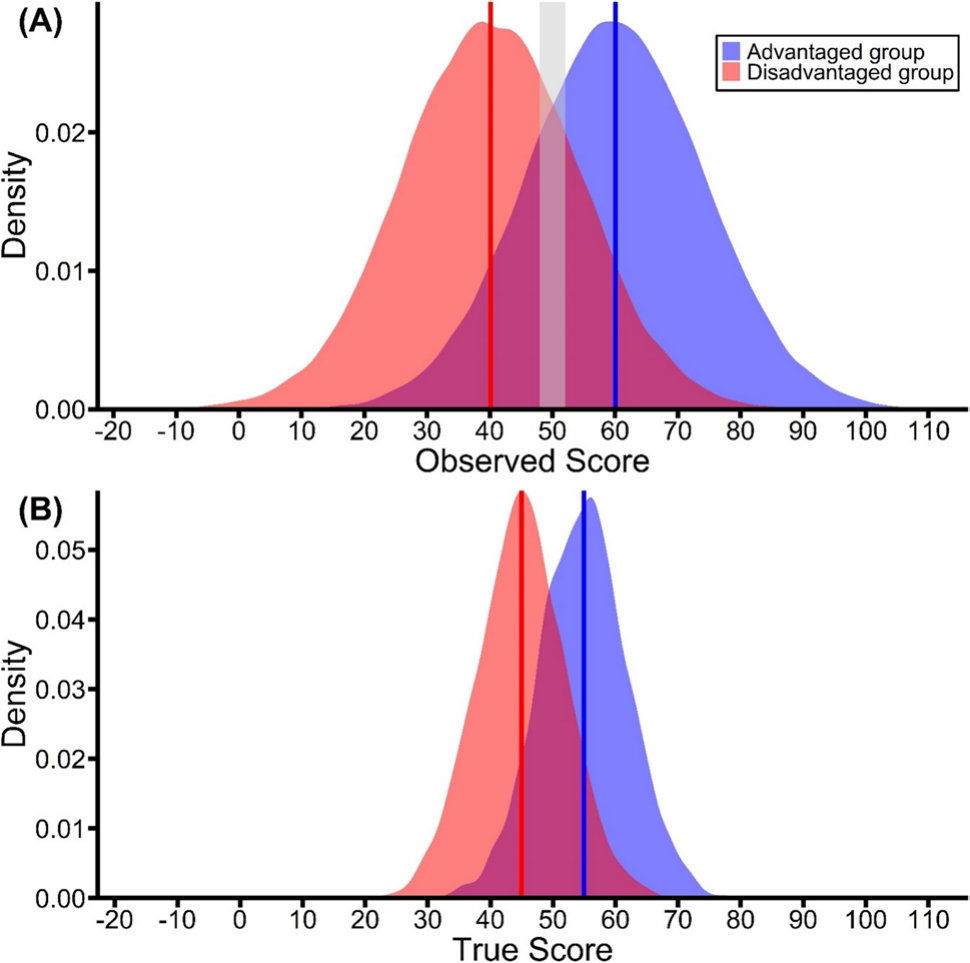
(**A**) Density distributions of observed scores. The vertical blue and red lines represent the observed score means for the advantaged and disadvantaged groups. The shaded area represents the selected *observed* scores between 48 and 52. (**B**) Divergence of true score means for participants falling within the shaded area in panel A. The vertical blue and red lines represent the *true* score means for the advantaged and disadvantaged subgroups

The simulated density distributions of observed scores for both the advantaged and the disadvantaged groups are illustrated in Fig. 1A. The means of the observed scores for the advantaged and disadvantaged groups are denoted by vertical blue and red lines, respectively. It is evident that the observed score means closely match the true score means for both groups. This is attributable to the zero-mean error terms in our model, which, barring sampling error, do not influence the overall means of the score distributions when translating true scores to observed scores.

Now we focus on subgroups composed of participants with observed scores within a specific range: do the true score means for these selected individuals (represented by the gray band in Fig. 1A) remain the same across both groups? Figure 1B unveils a surprising divergence. Contrary to expectations that true score means should be similar – given that error terms are zero-centered – the simulation shows otherwise. Among the participants selected from the constrained observed score range of 48–52, the true score mean for the disadvantaged subgroup is 44.94 (denoted by the vertical red line in Fig. 1B), while for the advantaged subgroup, it is 54.91 (represented by the vertical blue line). Importantly, this same basic pattern emerges wherever the interval is placed and whatever its width, including (as noted above) ones bounded by either -∞ or +∞. For instance, if the interval extends from -∞ to 30, the mean true scores are 30.87 and 42.70 in the disadvantaged and advantaged subgroups, respectively.^1^ An interval such as this is, of course, of particular relevance to the miss-CR contrast approach, which compares items drawn from two distributions and all of which fall below a criterion.

Why does such a divergence occur between the two subgroups despite the constraints set on observed scores? The fundamental statistical concept of RttM provides insight. RttM is a phenomenon rooted in statistical considerations and measurement error. To explicate this, recall that we simulated each observed test score based on two components: the individual’s true ability (or true score) and a random error inherent in the measurement process. Occasionally, this random error can cause observed scores to appear more extreme or more moderate than the true scores are (Khan & Olivier, 2019, 2023). Nevertheless, when we take a subsequent measurement, these errors, being random, do not always repeat in the same way. Instead, scores often “regress” or move closer to the group’s true mean on a subsequent test.

Crucially, as shown in Fig. 1B, the true score means for both selected subgroups shift towards their respective true score population means. This shift in true scores is modulated by their initial distance from the group-specific population mean. Specifically, when we select participants within a specific range of observed scores, these individuals’ scores are influenced by random error. This phenomenon can be understood through Eq. (1): although true scores ${T}_{i}$ and error components ${E}_{i}$ are uncorrelated, observed scores ${X}_{i}$ correlate positively with ${E}_{i}$ due to their shared term, ${E}_{i}$ itself. Consequently, higher observed scores ${X}_{i}$ are typically coupled with elevated ${E}_{i}$ values, and the opposite holds true for lower ${X}_{i}$ scores. When we subsequently evaluate the true scores across these two subgroups of participants, essentially removing random errors, the scores naturally shift toward their respective population means, underscoring the RttM phenomenon. These findings demonstrate Kelley’s Paradox. Specifically, although the selected disadvantaged participants exhibit observed scores comparable to those obtained by their advantaged counterparts, this does not necessarily signify higher potential, as one might assume. In fact, the resulting patterns unveiled in the true score means suggest that the selected disadvantaged participants likely harbor less potential than their advantaged peers.

**Fig. 2:**
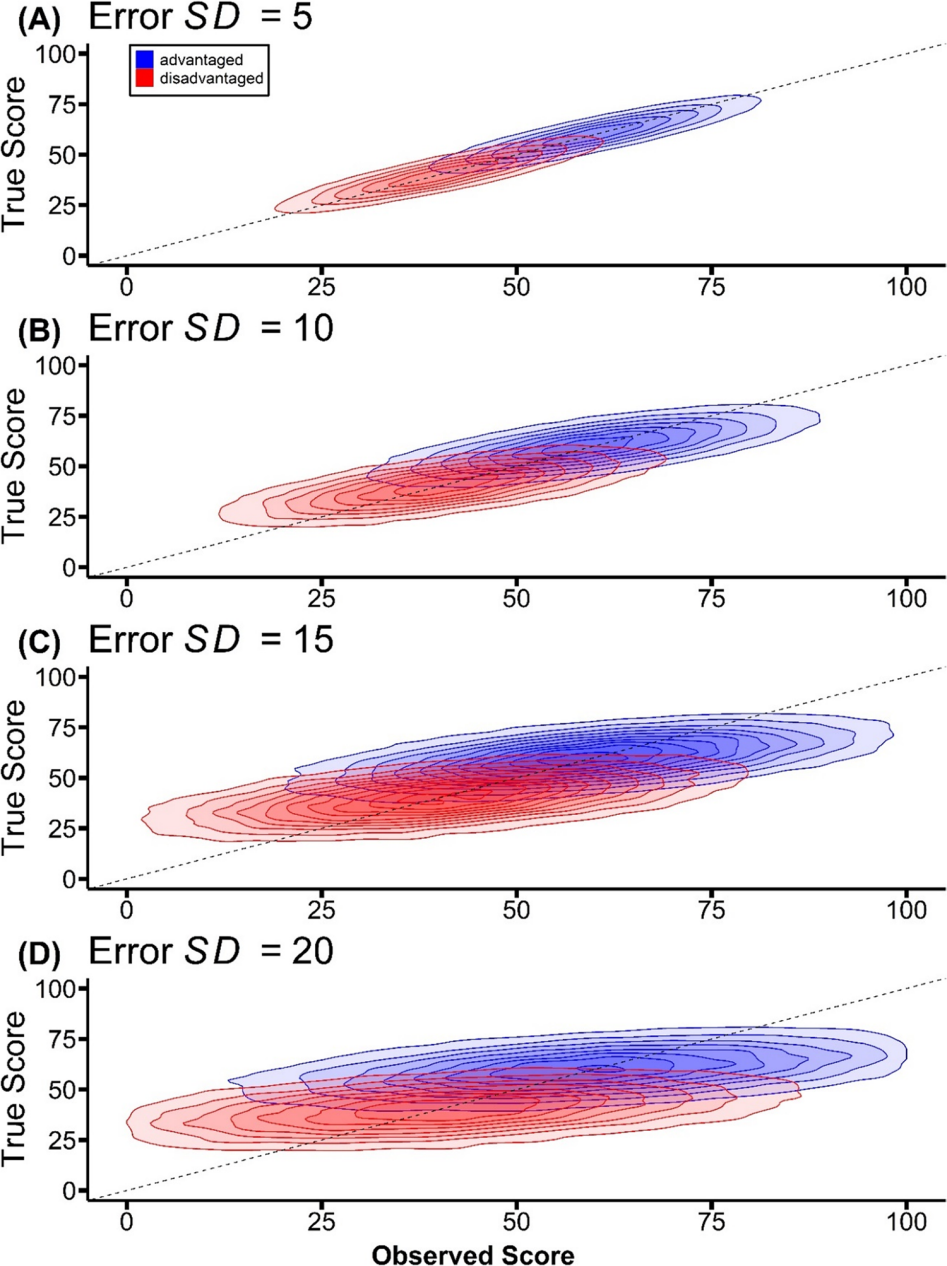
(**A**)-(**D**) Iso-probability curves of true and observed scores for advantaged and disadvantaged subgroups, at varying error *SD*s. The error *SD*s were set at 5, 10, 15, and 20 respectively, yielding reliabilities of 0.8, 0.5, 0.31, and 0.2. Contours represent lines of equal probability density, indicating regions where data points are equally likely to occur within the specific distributions of true and observed scores

Crucial to the analysis above is the inclusion of measurement error. One way to clarify the role of measurement error is via the concept of reliability. For the sake of simplicity, in the simulation above, true scores and error terms for both groups were sampled from normal distributions with variances of 100. According to classical test theory, the reliability of a measure is defined as follows:

4 $${\rho }_{{XX}^{^\prime}}=\frac{{\sigma }_{T}^{2}}{{\sigma}_{T}^{2}+{\sigma}_{E}^{2}}$$

where ${\rho }_{{XX}^{^\prime}}$ represents the reliability of the measure. *X* and *X' *represent two sets of observed scores obtained from the same individuals, which could be from random halves of a single multi-trial test, from the same test administered at two different times, or from parallel forms of the test administered at the same or different times. In light of the current parameter values, the simulated reliability of the score measure is 0.5. While this might be deemed low for many conventional applications, it is consistent with reliability estimates reported in studies on unconscious memory (Vadillo et al., 2022), and even general cognitive processes (Huber et al., 2019). Importantly, since the extent of RttM is contingent upon the reliability of the measure in question (Campbell & Kenny, 1999; Lee & Shanks, 2023; Rothkirch et al., 2022; Shanks, 2017) – as the greater the dispersion of these random errors, the more pronounced the RttM effect becomes (Barnett et al., 2005; Yaron et al., 2023) – Kelley’s Paradox intensifies with a less reliable measure. This is illustrated in the simulation presented in Fig. 2 on how changes in error magnitudes (and, by extension, reliability of the measure) impact the relationship between true and observed scores for simulated participants with observed scores across the range. This figure showcases four panels of iso-probability curves, each from a distinct iteration of the simulation. The iterations differ only in the *SD* of their error term distributions, ranging from 5 to 20, incremented by 5. The group means and true variances are as before, hence the true score *SD* = 10.

The relationship between error *SD* and the reliability of the measure is evident: reliability is at its peak (i.e., 0.8) when the error *SD* is set at 5, whereas reliability is the lowest (i.e., 0.2) when the error *SD* is set at 20. If we now select participants with a given score, some from the advantaged and some from the disadvantaged population, a striking pattern emerges. When error is low (and reliability high), the iso-probability contours are tightly clustered around the diagonal, suggesting that participants with the same measured score are likely to have similar true scores (top panel). In fact, in the hypothetical scenario where the error *SD* is set at 0, the iso-probability contours would collapse to the diagonal, indicating perfect reliability with no error, and observed scores would precisely match true scores. But as error increases (and reliability reduces), the contours spread further apart vertically, indicating that participants with the same measured score are more likely to diverge in their true scores (bottom panel) depending on whether they are drawn from the advantaged or disadvantaged population. Eventually, when *SD* = 20, the expected true score of a participant with an observed score of 50 is 58.29 if that participant is from the advantaged group but 41.61 if they are from the disadvantaged group, based on the simulated data set.^2^ Picking individuals or subgroups who appear to have identical scores leads us to be tricked: their true scores are in fact highly divergent. This trend reinforces the premise that Kelley’s Paradox is exacerbated by greater error variability and mitigated when the error *SD* is minimal.

Now consider a memory experiment where participants respond to various items during a test phase. Some items were previously presented to them (i.e., old items), while others are entirely new. By design, the true memory strength for new items should be zero, given that the participant has never encountered them. In contrast, old items are assumed on average to possess some positive true memory strength due to prior exposure. When tested on recognition memory of the items, there are four possible outcomes: correct identification of old items (hits), correct identification of new items (CRs), misidentification of old items as new (misses), and misidentification of new items as old (false alarms). In the miss-CR approach we focus on CRs and misses, since the participant exhibits no *observed* recognition memory for such items (i.e., these items are recognized as new), reasoning that any difference in another measure (behavioral or neural) between these two types of items is then viewed as evidence of unconscious memory.

But the discussion of Kelley’s Paradox above should alert us. Selecting items based on observed recognition memory is similar to selecting participants based on observed test scores. As all measurements are susceptible to errors, when a participant deems an item as new (falling below the decision criterion), its observed memory strength is likely to deviate from its true memory strength. Given the assumption that the true memory strength mean for old items is higher than that for new items, the true memory strength mean for selected old items is likely to be greater than that for new items. We believe we have selected items equated for conscious memory, but in reality we have not done so.

### Formal analysis based on Signal Detection Theory

The simulation described above (Fig. 1) clearly bears a close resemblance to SDT, in that samples are drawn from normal distributions along an underlying “strength” dimension. In this section we now develop an SDT-based analysis of the miss-CR contrast approach which takes us beyond the intuitive but simplistic example captured in the simulation. In addition to formalizing the conditions under which the true scores of samples of misses and CRs differ, and quantifying these differences, SDT also allows us to consider conditions in which the variances of the distributions are unequal.

We adopted the assumption of equal variance across true score distributions for the disadvantaged and advantaged groups in the discussion above. Yet, in analyses of recognition memory (where the miss-CR method has most often been employed) this assumption is typically found to be invalid. When SDT is fitted to empirical findings, and in particular to receiver operating characteristic (ROC) functions, the variance of the memory strength distribution for old items is usually estimated as being greater (by a factor of around 1.3) than that of new items (Lange et al., 2019; Rotello, 2017). Transitioning from simulations to a formal analytical exposition grounded in SDT, we aim to generalize our analysis to scenarios characterized by unequal variances. Hence the goal of this section is to demonstrate that the use of the miss-CR contrast approach to infer unconscious processing is strictly valid only under very limited conditions. Although the analysis is framed for simplicity in terms of recognition memory for old and new items, it applies equivalently for any discrimination between targets and non-targets, as in a perception experiment.

*T*_*M*_ and *T*_*CR*_ represent the true memory strength of misses and correct rejections, respectively. The assumption on which the miss-CR contrast approach operates can then effectively be boiled down to the specific case where *T*_*M*_ equals *T*_*CR*_, which, as we will see, only holds under very restricted conditions. Any deviation from this equality, and in particular any cases where *T*_*M*_ > *T*_*CR*_, indicates that observed differences in behavioral or neural metrics when comparing misses and correct rejections are not valid evidence for unconscious memory.

The following analytical approach rests on a derivation by Arnold et al. (1993), as elaborated in Appendix A. Essentially this equation estimates the expected true mean memory strength for items constrained within a truncated range, from a lower bound *C*_*l*_ to an upper bound *C*_*u*_, such as when decision criteria are set on the observed memory strength. In particular, when we focus on high-confidence CRs and misses, the range is from *C*_*l*_ = -∞ to a variable criterion, *C*_*u*_. We assume that true memory strengths of old and new items are each normally distributed, with new items centered at a mean of 0 and a *SD* of 1 and the old item mean and *SD* being free to vary. Normally distributed error, centered at 0 and with *SD*s ranging from 0 to 2, is added as measurement noise to the true memory strengths.^3^ We also examine the impact of two distinct decision criteria (*C* = 0 and -1) reflecting the strength signal below which an item is classified as “new” – resulting in CRs in the case of new items and misses in the case of old items.

**Fig. 3:**
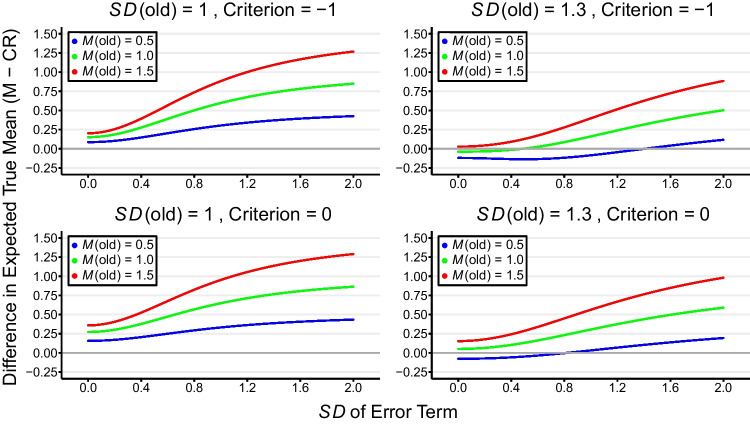
Differential expected true means in memory strength between misses and correct rejections as a function of error *SD*s across various conditions. Each panel corresponds to distinct combinations of *SD*s of old items (1 and 1.3) and decision criteria (-1 and 0). *SD*(new) is set to 1 in all panels. The color-coded lines represent true mean memory strengths (0.5, 1.0, and 1.5) set for old items

Figure 3 illustrates the expected mean difference in true strength between misses and correct rejections as a function of the *SD* of the error term under different distribution parameters. The blue, green, and red curves within each of the four panels correspond to true mean strengths of old items set at values of 0.5, 1.0, and 1.5, respectively. An assumption of equal variances for the old and new item distributions is shown in the left panels, while an *SD* of 1.3 represents unequal variances (right panels).

A pronounced upward trajectory across all conditions – especially marked at higher true mean values for old items – indicates that as the error variance increases, the extent to which the expected memory strength for misses exceeds that for correct rejections amplifies. Crucially, intersections of the curves with the y-axis’s zero point, denoting identical expected true mean memory strength for misses and CRs (i.e., *T*_*M*_ - *T*_*CR*_ = 0), are rare. This state of equilibrium, critical for validating claims of unconscious memory when differences in other behavioral or neural measures are observed, manifests solely with unequal variances and when the true mean of old items is relatively low (i.e., 0.5 and 1).

Under some parameter settings (e.g., unequal variances and the old item and error means being low) the true mean for old items (misses) is less than that for new items (CRs) (Berry & Shanks, 2024). It might be assumed that the miss-CR contrast approach is valid under these conditions. However, if the implicit measure is a neural signal which is larger for misses than CRs, an alternative possibility is that there is a negative association between the neural signal and memory strength, as often observed in phenomena such as repetition suppression (Lee et al., 2020). Even in the complete absence of unconscious memory, memory strength and neural activity may be inversely correlated. All in all, so long as there is a difference in true mean memory strength between misses and correct rejections, it is difficult to infer unconscious memory based on the miss-CR contrast approach.

### Strength skewness and the *miss-CR contrast* approach

The curves shown in Fig. 3 display another important property that demonstrates a second factor at play in addition to measurement error. The miss-CR difference is predicted to often be non-zero *even when error is zero*. Thus, under conditions in which regression to the mean cannot be relevant, misses and CRs are not expected to have equal mean strength. The fundamental assumption of the miss-CR contrast approach, that misses and CRs have equal strength, is invalid *even under standard SDT*.

**Fig. 4:**
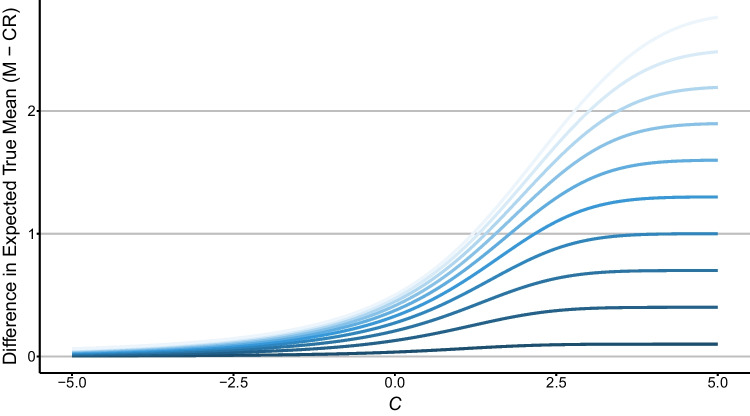
Differential expected true means in memory strength between misses and correct rejections as a function of decision criteria (i.e., *C*) across different *d′* values, assuming equal variances for old and new item distributions. Each of the ten curves represents a unique *d′* value, ranging from 0.1 (the lowest curve) to 2.8 (the highest curve)

Why does SDT make this prediction? Consider first the case where the variances of the distributions are equal (left panels of Fig. 3; *SD*(error) = 0). Within any given interval on the strength dimension, including one from -∞ to *C*_*u*_, the distribution of old item strengths is always more skewed towards the left (i.e., negative skew) than the distribution of new item strengths. The mean strength of misses is always greater than that of CRs falling within the same interval on the strength dimension (and this does not depend on the placement of the criterion). Skewness is simply a measure of asymmetry, with negative skew meaning that more of the mass is to the right of the median. Hence mean strength is always greater for misses than CRs as reflected in the left panels of Fig. 3 (in Appendix C, we provide a more detailed explanation of strength skewness for interested readers). This is more explicitly illustrated in Fig. 4, where the variances of old and new item distributions are both set at 1, while the ten blue curves represent different values of *d′* (0.1, 0.4, 0.7, 1.0, 1.3, 1.6, 1.9, 2.2, 2.5, 2.8, from dark to light). Like Fig. 3, Fig. 4 shows the difference in the mean strengths of misses and CRs in the interval from -∞ to *C.* The mathematical derivation on which this is based (a special case of the equation for Fig. 3) is given in Appendix B. Crucially, in all cases the difference is greater than zero. Indeed, if *d′* is large then the difference between the mean strengths of misses and CRs can also be large, especially if the criterion *C* for responding “old” is highly conservative.

Now consider the case in which the variance of the old item distribution is greater than that of the new item distribution (right panels of Fig. 3; *SD*(error) = 0). In this case the old item distribution in the relevant interval may be more right-skewed, more left-skewed, or equal to the new item distribution, depending on the precise parameters. Hence, as shown in Fig. 3, the miss-CR difference can be negative, zero, or positive.

In sum, there is not one but two factors that entail that the fundamental and indispensable assumption of the miss-CR contrast approach is usually invalid. The first of these is regression-to-the-mean occurring when item strengths include error (Kelley’s Paradox) and the second is the inequality of old and new items falling in an interval on the strength dimension (strength skewness). These are independent and additive factors that contribute to the overall difference in strengths for misses and CRs. Note, however, that the impact of the first factor will usually be greater than that of the second factor: the miss-CR difference increases sharply as error increases in Fig. 3. While the impact of Kelley’s Paradox is always in the one direction (as error increases the miss-CR difference always increases), strength skewness can lead to CRs having greater mean strength than misses or the opposite (as in the bottom right panel of Fig. 3).

Also noteworthy is that noise becomes relatively more important as the width of the interval narrows. As this width approaches zero, the mean strengths of old and new items converge. This could have practical implications if the relevant awareness measure can be shown to be highly reliable (with error hence being small). If the misses and CRs are derived from a narrow interval (e.g., between criteria defined as *absolutely certain the item is new* and *fairly certain the item is new*), then the miss-CR contrast approach would become approximately valid.

### The prevalence of the *miss-CR contrast* approach

In this section, we offer a non-exhaustive literature review highlighting the widespread adoption of the miss-CR contrast approach in unconscious processing research. It is important to note that terminology such as “misses” and “correct rejections” is not uniformly applied across studies, and their operational definitions can vary contingent on the research context. Given these inconsistencies, our literature review is narrative in nature. Nevertheless, this review emphasizes the prevalence of the miss-CR contrast approach across a broad range of fields and research questions, even though the extent to which this analytic approach has influenced particular conclusions varies from one study to the next.

Table 1 showcases studies from high-impact journals including *Nature* and *Journal of Experimental Psychology: General*, attesting to versatility of the miss-CR contrast method. Researchers have used this approach in conjunction with diverse methodologies, from electroencephalogram (EEG) assessments (Addante, 2015; Addante et al., 2023) to behavioral response time measures (Sheldon & Moscovitch, 2010). The studies also employ a wide range of stimuli including words (Woollams et al., 2008), faces (Lehmann et al., 2004), and line drawings (Kark et al., 2016, 2020). The collection of studies also confirms the adoption of the miss-CR contrast approach across diverse areas, from facial imitation (Arias et al., 2018), to emotion processing (Jaeger & Rugg, 2012), visual recognition memory (Thakral & Slotnick, 2015), somatosensory processing (Grund et al., 2021), and brain stimulation (Grund et al., 2021). Dependent measures are both behavioral and neural.

**Table 1:** Studies from high-impact journals including *Nature* and *Journal of Experimental Psychology: General* attesting to versatility of the miss-CR contrast method

Study	Method	Stimuli	Relevant finding
Addante (2015)	EEG	Old and new words	Control subjects’ ERPs were more positive for old items (misses and neutral responses combined) than new items (correct rejections and neutral responses combined), reflecting implicit memory
Addante et al. (2023)	EEG	Old and new words	More positive ERPs for misses than for correct rejections, reflecting implicit memory
Arias et al. (2018)	EMG	Transformed and non-transformed spoken sentences	Greater zygomatic activity for misses than correct rejections, reflecting unconscious recognition of auditory smiles
Gomes et al. (2015)	Eye-tracking	Old and new images of common objects	Differential pupil dilation between misses and correct rejections, reflecting unconscious object memory
Grund et al. (2021)	fMRI	Trials with and without electrical stimulation	Greater neural activity for confident misses than confident correct rejections, reflecting non-conscious stimulus processing
Henson et al. (2005)	fMRI	Old and new words	Implicit memory operationalized as difference in neural activity between misses and correct rejections
Jaeger and Rugg (2012)	EEG	Old and new pictures of objects	Greater neural activity for misses than correct rejections in emotional contexts, reflecting implicit memory retrieval
Kanai et al. (2010)	Behavioral performance	Visual targets	The comparison of the rate of high-confidence correct rejections with that of high-confidence misses was used to index subjective discriminability of invisibility, which in turn was used to differentiate between perceptual and attentional blindness
Kark et al. (2016)	fMRI	Old and new line drawings	Differential neural activity between correct rejections and misses, reflecting implicit repetition suppression and repetition enhancement effects
Kark et al. (2020)	fMRI	Old and new line drawings	Differential neural activity between correct rejections and misses, reflecting long-term implicit repetition suppression and repetition enhancement effects
Lehmann et al. (2004)	fMRI	Old and new photos of adult faces	Greater neural activity for misses than correct rejections, reflecting unconscious discrimination of stimuli
Marois et al. (2004)	fMRI	Images of scenes and scrambled scenes (i.e., no scene)	Greater neural activity for misses than correct rejections, reflecting unconscious scene perception
Ramey et al. (2020)	Eye-tracking	Images of old and new scenes	Unconscious memory operationalized as difference in eye movement dispersion between high-confidence misses and high-confidence correct rejections
Ramey et al. (2019)	Eye-tracking	Images of old and new scenes	Lower scanpath ratio for high-confidence misses than high-confidence correct rejections, reflecting unconscious memory
Rugg et al. (1998)	EEG	Old and new words	More positive ERPs for misses than for correct rejections, reflecting unconscious memory
Sheldon and Moscovitch (2010)	Behavioral performance	Old and new words	Faster RTs for misses than for correct rejections, treated as implicit priming
Slotnick and Schacter (2004)	fMRI	Old and new shapes	Greater neural activity for misses than correct rejections, reflecting implicit repetition priming
Slotnick and Schacter (2010)	EEG	Old and new shapes	Greater neural activity for misses than correct rejections, reflecting nonconscious priming
Stark and McClelland (2000)	Behavioral performance	Old and new words and pseudowords	Faster RTs for misses than for correct rejections, reflecting implicit repetition priming
Thakral and Slotnick (2015)	EEG and fMRI	Old and new shapes	Greater neural activity for misses than correct rejections, reflecting nonconscious processing
Woollams et al. (2008)	EEG	Old and new words	More positive ERPs for misses than for correct rejections, reflecting implicit repetition priming

While some studies adopted simple binary awareness measures to categorize test items as misses and CRs (e.g., Jaeger & Rugg, 2012), others incorporated more fine-grained measures to gauge different levels of awareness. For instance, a 6-point recognition response measure was utilized in Ramey et al. (2019) to assess awareness. After viewing a test item, participants were prompted to respond using a scale where 1 = *I’m sure it’s new*, 2 = *Maybe it’s new*, 3 = *I don’t know*, 4 = *Maybe it’s old*, 5 = *I’m sure it’s old*, and 6 = *Recollect old*. To infer unconscious memory, the authors focused on the contrast in other eyetracking measures between old images that received a rating of 1 (i.e., misses) and new images that also received this rating (i.e., CRs). This approach aimed to minimize potential interference from conscious memory (for a detailed analysis of this study, see Lee & Shanks, 2023). Regardless of how misses and CRs were operationalized or the complexity of the awareness measure, the core premise of the miss-CR contrast approach remained the same: items were first classified and selected to represent misses and CRs, and subsequent contrasts were drawn based on other performance metrics or attributes associated with these two types of items. In two of these studies, no significant difference between misses and correct rejections was revealed on the key behavioral/neural dependent measure, thus the authors concluded that they failed to find implicit repetition suppression (Henson et al., 2005) or that dispersion of viewing was influenced by unconscious memory (Ramey et al., 2020). Obviously these null results are unlikely to be compromised by regression to the mean or strength skewness.

To further illustrate the application of this approach, we draw upon several illustrative examples. To begin with, we refer back to Rugg et al.’s (1998) study, previously cited in the Introduction. This study posited that unconscious memory can be revealed through differences in neural activity between new words (i.e., CRs) and misclassified old words (i.e., misses). Beyond its substantial influence as indicated by citations (> 900 on Google Scholar in October 2023), the miss-CR contrast approach Rugg and colleagues adopted has garnered widespread recognition in influential outlets. Take, for example, *Principles of Neural Science*, a widely adopted neuroscience textbook. In the latest edition (6th Ed.) of the textbook, the Nobel laureate Eric R. Kandel and his colleagues (2021) referenced Rugg et al. (1998), elucidating on how unconscious memory can be demonstrated via the miss-CR contrast approach:

A widely used protocol tests subjects’ ability to recall lists of words they have memorized, a task that taps a form of declarative memory. In the recall phase, a subject is presented with a list of the words that were on the study list plus new words. An amnesic patient has great difficulty with this type of task and may misclassify most of the previously seen words as new since she cannot recall seeing them before. Nevertheless, the brain activity elicited by reading old words is different from that elicited by the new words: There is unconscious recognition of a difference, equivalent to that shown by patients with unilateral neglect or prosopagnosia. Normal subjects usually find this task easy, but they too will occasionally misclassify old words as new; as with amnesiacs, evoked brain responses in normal subjects register the distinction lost to conscious recall (Chapter 59).

Gomes et al. (2015) provides another instance of the application of the miss-CR contrast approach. In their investigation, the authors contrasted pupil dilation responses between misses (termed “Ms” in their article) and CRs, after controlling for associated reaction times. Gomes et al. (2015) explained this as follows:

Even though RT-matched Ms showed larger pupil dilation than CRs, we used the nonmemory RT confound matching procedure… to ensure that this effect reflected unconscious object memory rather than a failure to match for nonmemory-related difficulty of the pictures selected as Ms and CRs… Therefore, the familiarity level of Ms must have been effectively at chance and the enhanced pupil dilation of Ms must have reflected unconscious object memory of some kind rather than an effect of above chance, but below threshold levels of familiarity (p. 761).

Similarly, Grund et al. (2021) inferred unconscious tactile stimulus processing by contrasting neural correlates of misses and CRs. According to the authors:

By comparing the contrast of undetected stimuli to correctly rejected catch trials, neural processes associated with non-conscious stimulus processing of near-threshold stimuli can be assessed (p. 2).

In a more recent study by Addante et al. (2023), neural correlates associated with misses and CRs were explored. Participants’ recognition of stimulus words was recorded using a 5-point recognition confidence scale. While their methodology echoed the conventional approach of Rugg et al. (1998), Addante et al. (2023) introduced a nuanced adjustment. Drawing from a procedure devised by Woodruff et al. (2006), Addante et al. (2023) ensured an equal number of randomly selected trials for both old and new words within each level of recognition confidence. This strategy aimed at balancing the memory strength of old and new items within each response category, thereby permitting a more rigorous comparison of misses and CRs uncontaminated by residual explicit memory. As Addante et al. (2023) explicated:

By virtue of the shared reported strength of memory responses comprising both the old and new ERP conditions, this procedure eliminated the possible confound discussed above for memory misses: that they might reflect differential amounts of memory strength, or be contaminated by sub-threshold explicit memory difference among old and new conditions. This new method of measuring ERPs of implicit memory was thus stronger and more precise than methods used in prior studies (i.e., generic measures of miss vs. correct rejections), and was presumed (though not tested) to be free of conflation with other variables such as explicit memory (p. 3).

Finally, as previously discussed, Ramey et al. (2019) focused on contrasts between high-confidence misses and CRs (i.e., old and new items rated 1) to infer unconscious memory. Their rationale was that this stringent criterion “ensured that none of the scenes used in the unconscious memory contrast were contaminated by conscious recollection or familiarity” (p. 74). An overarching theme emerges from these examples: regardless of how the miss-CR contrast approach is framed and operationalized, the memory strength for both item categories was consistently deemed equivalent, and thus any differential outcomes in other indirect variables of interest were interpreted as evidence of unconscious memory.

This brief review serves to underline both the scale and diversity of applications of the miss-CR contrast approach. Another crucial observation is the absence of any acknowledgment of the double-dipping problem and Kelley’s Paradox – or more broadly, RttM – by researchers in these studies, despite the widespread warnings raised by statisticians (Campbell & Kenny, 1999; Wainer, 2000; Wainer & Brown, 2006). We are not suggesting that all findings employing the miss-CR contrast approach – thus susceptible to Kelley’s Paradox and strength skewness – are categorically invalid. In numerous instances, conclusions were grounded in a range of evidence and varied analyses. It is even possible that the problems highlighted in the sections above are less relevant to some domains than others. For example, as Fig. 4 shows, strength skewness has a larger impact on the difference in the mean strengths of misses and CRs as *d′* increases. In studies of recognition memory, *d′* is typically well above zero, whereas in studies on unconscious vision, stimulus visibility is often manipulated to yield near-threshold perception (*d′* ≈ 0, e.g., via backward masking). Thus, it is possible that strength skewness is less of an issue when the method is employed in the latter than the former domain.

Nonetheless, when conclusions rest predominantly on the miss-CR contrast method, their logical underpinnings are inherently susceptible to challenge. To put it another way, it is only via a detailed case-by-case assessment that the scale of the problems can be determined in a given application. For each example in Table 1, it is conceivable that a model exists – one that does not distinguish between conscious and unconscious processes at the latent level – but which can nevertheless predict the critical differential pattern as a manifestation of Kelley’s Paradox and/or strength skewness (for instance, see Lee & Shanks, 2023). The varying performance in metrics apart from awareness, between misses and CRs, might arise purely from the fact that the method does not in fact equate conscious mental states. The degree to which such models could account for specific observed effects or the entirety of response nuances necessitates individual scrutiny, but it is clear that conclusions failing to address Kelley’s Paradox and strength skewness lack robustness.

Advocates of the miss-CR contrast method might counter that Kelley’s Paradox is only a problem if the reliability of their awareness measures is low. As revealed in the simulations reported above (Fig. 2), the degree of divergence in true scores of subgroups matched for their observed scores is a function of the magnitude of error, and hence of the reliability of the measure. Indeed, the divergence is modest if reliability is fairly high (0.8). So, what evidence do we have that the reliability of the awareness measures employed in the studies in Table 1 is sufficiently low to render Kelley’s Paradox a serious concern?

It is very rare for studies to report this metric. However, reliability values have been calculated for a reasonable sample of representative studies. Yaron et al. (2023) reported the Spearman–Brown-corrected reliability of awareness measures in 18 experiments and found that it exceeded 0.7 in only four. Strikingly, in 9/18 (50%) of them it was either negative or close to zero. When reliability is near zero, the expected true scores of subgroups matched for their observed scores regress completely to the group means. Thus, if an experiment employs an entirely unreliable recognition confidence measure and applies the miss-CR approach to items selected from the lowest confidence category, the true scores of the misses and CRs will be at the overall old and new item means, which of course will be different unless *d*′ = 0. Rothkirch et al. (2022) reported reliabilities of awareness measures used in 12 implicit learning and unconscious processing studies. Only three were above 0.8.

Of course, it is possible that some of the experiments in Table 1 employed awareness measures with high reliability. This is certainly the case in Ramey et al.’s (2019) experiment, for which the reliability of their recognition confidence scale was 0.87 (Lee & Shanks, 2023). In our experiment reported in the next section, the mean Spearman–Brown-corrected split-half reliability of the single-item recognition task was 0.78, based on 5,000 random splits, indicating that the recognition task was also of adequate reliability. But even in these cases there is appreciable error, and it is only by detailed modeling that we can gauge whether the magnitude of this error is sufficient to explain the divergence seen in the implicit measure (eye movements in this case) – which is precisely what Lee and Shanks (2023) demonstrated. For all the studies adopting the miss-CR contrast approach for which the reliability is unknown, the authors’ conclusions rest on an assumption (${\rho }_{XX^{^\prime}}$ = 1.0) that is known to be empirically false.

And of course, our analyses above show that even when there is no error (reliability ${\rho }_{XX^{^\prime}}$ = 1.0), the miss-CR difference will almost always be non-zero as a result of the second factor, strength skewness.

## Experiment

Following our previous simulations and discussion on how Kelley’s Paradox and strength skewness can be linked to the miss-CR contrast approach, we now report an experiment to test the hypothesis that high-confidence recognition misses and correct rejections usually do not have equal true memory strengths. As we expected overall recognition *d*' to be fairly high (see below), we predicted any negative effect of differential strength skewness to be minimal and hence the miss-CR difference would be positive (see Fig. 3). In this experiment, after studying a word list, participants completed a single-item recognition test in which they made old/new judgments to studied and new words on a recognition confidence scale, followed by a two-alternative forced-choice (2AFC) test in which the key trials paired high-confidence misses and correct rejections from the single-item stage (Lee & Shanks, 2023, recently adopted a similar approach in the context of implicit learning).

We hypothesized that the overall accuracy for judging old words correctly in the 2AFC task would be significantly above chance. More crucially, in the same task, for pairs comprising old words falsely judged new with high confidence (i.e., high confidence misses) and new words judged new with high confidence (i.e., high confidence correct rejections), we expected that the accuracy of selecting old words correctly would be significantly above chance.

The experiment was preregistered on the Open Science Framework (https://osf.io/pk6an).

### Methods

#### Participants

An a priori analysis using G*Power (version 3.1.9.7; Faul et al., 2007) was conducted before data collection, which indicated that a sample of 71 participants would be needed to detect a small effect (*d*_*z*_ = 0.3) in a one-sample *t*-test with a power of .80 at a one-tailed alpha level of .05. We aimed to reliably detect the difference between the accuracy of selecting old words correctly in high-confidence miss/correct rejection pairs and chance performance (i.e., an accuracy of 0.5) in the 2AFC task. A total of 75 participants located in the UK (35 males and one non-binary; *M*_*age*_ = 34.12 years, *SD*_*age*_ = 10.30, range = 18–59 years) were recruited via Prolific. All participants had normal or corrected-to-normal vision and were asked to complete the experiment via a web browser in a quiet environment without distractions. None of the participants took part in previous related experiments. Informed consent was obtained from all participants, and they were paid £4 in exchange for completion of the experiment. This study was approved by the UCL Research Ethics Committee.

#### Materials

The experiment was programmed with PsychoPy (Peirce et al., 2019). For each participant, two sets of 60 words and one set of 20 words were randomly sampled without replacement from a list of 180 words. The first set was used as the words to be studied in the first task and as the old words in the single-item test; the second set was used as new words in the single-item test; and the last set was used as truly new words in the 2AFC test. The word list was selected from a normative pool of 1,200 words developed by VanArsdall and Blunt (2022). In the selection process, we first filtered out words above 1 *SD* or below -1 *SD* on the concreteness, familiarity, imagery, valence, and arousal ratings from the word pool. Subsequently, 180 words were randomly selected from the remaining 304 words, irrespective of their living/nonliving ratings, to form the word list to be used in the current study.

#### Procedure

The experiment, consisting of four tasks, was implemented on the Pavlovia website (pavlovia.org). Participants were allowed to run the experiment on any device of their choice, barring mobile devices. The four tasks were the study task, the distractor task, the single-item recognition judgment task, and the 2AFC task, respectively. Participants first completed a word classification task of 60 trials, serving as the study task. A word was presented in each trial, and participants’ task was to decide whether it referred to a living or nonliving object as quickly as possible by pressing either the “left” or “right” arrow key. The order of presentation of words was randomized. No indication of a subsequent memory test stage was provided. Participants were given a short break upon completion of the study task.

Afterwards, participants completed a jigsaw puzzle distractor task. Each puzzle comprised nine pieces of an image, randomly arranged over the display. Participants moved the pieces using the mouse to form the complete image. After completing a puzzle, they pressed the spacebar to see a new puzzle. The distractor task lasted 5 min, before participants took another short break upon completion.

Then, participants completed a single-item recognition judgment task. A word was presented in each trial. The word was either one that appeared in the study task (i.e., an old word) or a new word. Participants reported whether or not they thought the word was presented in the study task based on a 4-point recognition confidence scale, with values 4 (“I’m sure it’s old”), 3 (“Maybe it’s old”), 2 (“Maybe it’s new”), and 1 (“I’m sure it’s new”). There were 120 trials (60 old words and 60 new words) in this phase, and the words were randomly ordered anew. A short break ensued after the end of this task.

Finally, the fourth task comprised a 2AFC recognition task. A pair comprising an old (i.e., studied) and a new word were presented side-by-side in each trial. Participants were given as long as they needed to select the word that had appeared in the study task (i.e., the old word) by pressing either the “left” or “right” arrow key. In 40 trials, the pairs included a new and an old word receiving the same recognition rating in the single-item recognition judgment task whenever possible. In cases where there were old and new words left unpaired because there were not enough words of each type given the corresponding rating, these words would be matched randomly, such that each pair consisted of old and new words receiving different ratings (i.e., “unequal pair”). A further 20 pairs were included which comprised studied and truly new words (i.e., words that had never appeared in the previous phases). These pairs were created by replacing words that were new in the single-item recognition phase in the following order: new words in unequal pairs were replaced first, then new words in pairs with equal ratings of 4, followed by new words in pairs with equal ratings of 3, and so on. As a result of the pairing process, the 2AFC task comprised a total of 60 trials. The sequence of word pairs appearing in this task as well as positions of old and new words across trials were randomized. Subsequently, participants were thanked and debriefed at the end of the experiment.

#### Data pre-processing

Following the pre-registration, to ensure that only those participants who were sufficiently attentive throughout the experiment were included in the statistical analyses, two exclusion criteria were adopted. First, we excluded participants who failed to achieve 85% accuracy in their living/nonliving judgments in the study task. Since some words in the word list were ambiguous regarding the type to which they belonged, we only focused on words that are clearly living or nonliving. Specifically, we deemed words above the living/nonliving rating of 600 as clearly living, whereas words below the living/nonliving rating of 200 were deemed as clearly nonliving. There were 138 clearly living or nonliving words in the word list. The living/nonliving rating ranged from 100 to 700. Second, we also excluded participants who failed to achieve 60% accuracy in selecting the old word correctly in the 20 pairs involving truly new words in the 2AFC task. In total, five participants were excluded, leaving a final sample of 70 participants in the subsequent analyses. In addition, for the critical one-sample *t*-test comparing the accuracy of selecting old words correctly from high-confidence miss-correct rejection pairs (i.e., word pairs rated 1) in the 2AFC task against chance performance, we excluded participants who had fewer than three valid pertinent trials. After applying this filter, the analysis was based on data from 29/70 (41%) participants. All the statistical analyses were carried out in R (R Core Team, 2022).

### Results

#### Study task

In general, participants were able to correctly judge clearly living or nonliving words with very high accuracies (*M* = .95, *SD* = .03). Conversely, when the whole study list was taken into account, words judged as living were significantly higher on the living/nonliving ratings (*M* = 602.84, *SD* = 36.56) than words judged as nonliving (*M* = 190.99, *SD* = 30.54), *t*(69) = 92.67, *p* < .001, *d*_*z*_ = 11.16. The results indicate that the participants understood the instructions correctly and were attentive during the study task.

#### Single-item recognition task

Collapsed across participants, the percentages of old words receiving ratings of 1–4 were, respectively, 6.64%, 8.50%, 11.45%, and 73.40%; in contrast, for new words, the percentages were, respectively, 48.71%, 30.29%, 11.90%, and 9.10%. These distributions suggest that, as expected, old words were given higher recognition confidence ratings compared to new words. In an exploratory analysis, we further probed participants’ performance in terms of *d'* scores. Specifically, old words correctly judged as old (i.e., responses receiving a confidence rating 3 or 4) were deemed as hits, while new words erroneously judged as old (by the same token, responses receiving a confidence rating 3 or 4) were deemed as false alarms. A mean *d'* score of 2.03 was revealed, suggesting that participants in general were able to make correct recognition judgments in this task. This confirms that negative strength skewness (that is, CRs > misses in strength) is unlikely to be relevant and that our key hypothesis – that misses would be selected more frequently than CRs in the forced-choice test – is reasonable. The decision criteria *C*_*1*_ - *C*_*3*_ (for instance *C*_*1*_ is the criterion below which participants responded with a rating of 1) were 0.03, 0.75, and 1.20, respectively. These were computed via maximum likelihood estimation, assuming equal variances, and note also that these criteria are with respect to the mean of the new item distribution (0) for consistency with Fig. 3 and 4.

#### 2AFC task

**Fig. 5:**
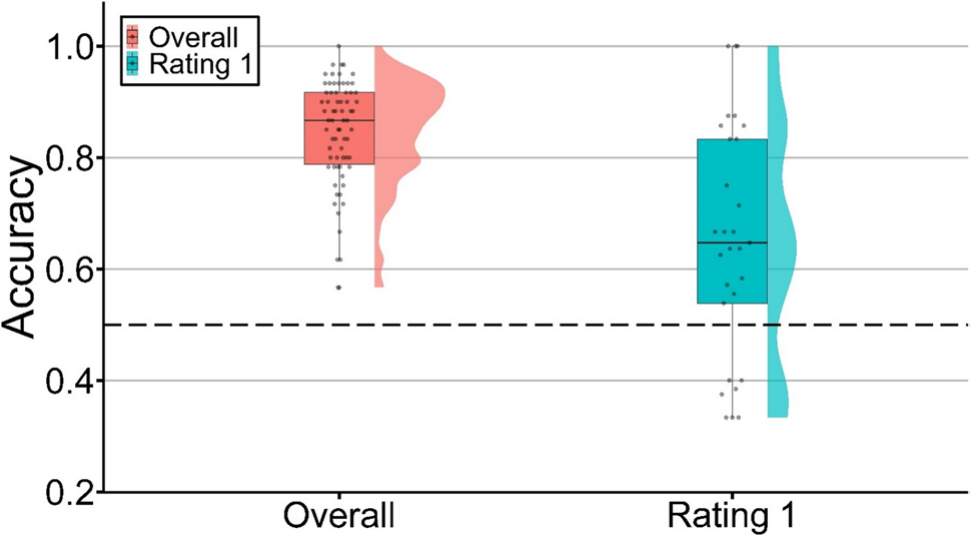
Respective distributions of mean overall accuracies and mean accuracies for trials rated 1. Each point represents a participant’s mean accuracy in selecting old words correctly. The dashed line represents accuracy at the chance level (i.e., 0.5)

The variable of interest was the mean accuracy for selecting the old word correctly across pairs of words in this task. Specifically, mean accuracy was defined as the proportion of trials in which participants correctly selected the old word. As a pair of old and new words was presented to participants in each trial, the chance accuracy is 50%. Across 60 trials in total, participants exhibited high overall accuracy (*M* = 84.40%, *SD* = 9.72%), significantly above chance, *t*(69) = 29.61, *p* < .001, *d*_*z*_ = 3.56. More critically, for word pairs receiving a rating of 1 from the single-item recognition stage (i.e., pairs comprising high confidence misses and correct rejections), participants on average also showed mean accuracy (*M* = 65.34%, *SD* = 21.06 %) significantly higher than chance, *t*(28) = 3.92, *p* < .001, *d*_*z*_ = 0.74. Figure 5 illustrates the respective distributions of mean accuracies.

In light of the large number of participants (41 of 70) excluded from the above analysis, we fitted a generalized mixed-effects model (GLMM) in an exploratory analysis. Before running the analysis, we excluded trials that paired words of different ratings, as well as trials involving truly new words, to ensure that the model was estimated based on word pairs with the same ratings. The GLMM allows us to include all participants, even those with missing data for some ratings.

Specifically, we fitted the GLMM with rating as a fixed effect and participant as a random effect, using the “lmerTest” package in R (Kuznetsova et al., 2017).^4^ We set the rating of 1 (“sure new”) as the baseline condition, such that the intercept represents the logit-transformed accuracy for selecting old words rated 1. The model was estimated using maximum likelihood estimation with Laplace approximation. The intercept was revealed to be significantly above 0, *b* = 0.60, *SE* = 0.15, *z* = 4.05, *p* < .001, which corresponds to an accuracy of correctly selecting the old word rated 1 of 0.65, 95% CI [0.58, 0.71]. This result corroborates the pre-registered analysis above, providing evidence for above-chance recognition of old words that had been previously rated as “sure new.” Here the effect is not restricted to a subset of the entire sample.

**Fig. 6:**
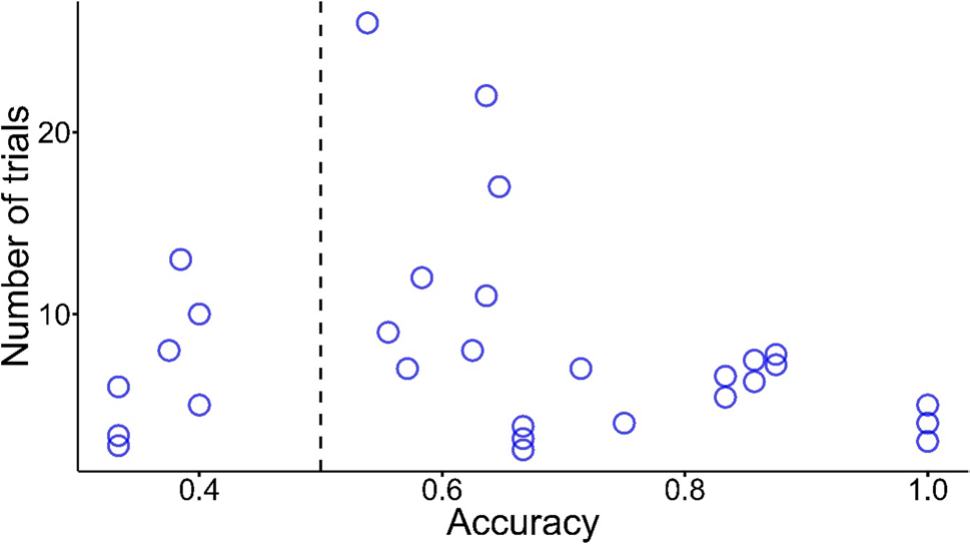
Distribution of trial numbers across different accuracies. Each point represents a participant’s mean accuracy with the corresponding number of trials encountered. Overlapping points have been vertically jittered to avoid overlap. The dashed line represents accuracy at the chance level (i.e., 0.5)

For the analysis on the word pairs rated 1 only, participants exhibited considerable between-subjects differences in both the mean accuracy and the number of trials encountered (*M* = 8.07, *SD* = 5.58). In light of this, as another exploratory analysis, we investigated whether there were systematic variations between participants’ mean accuracy and number of trials encountered in this task. As illustrated in Fig. 6, there was no apparent association between mean accuracy and number of trials encountered. This was further corroborated by the non-significant correlation between the two variables, *r*(27) = -.18, *p* = .35, 95% CI [-.51, .20].^5^

Finally, a possible objection to our key claim would be to suggest that the 2AFC task employed here is more sensitive than single-item recognition to unconscious memory. Such a view would propose that high-confidence misses and correct rejections are equated in terms of conscious memory (as routinely presupposed by those who employ the method) but not unconscious memory, and that 2AFC is sensitive to the latter. However, Lee and Shanks (2023) showed that the results are very similar when the 2AFC test is replaced by a yes/no final recognition stage: ratings were significantly higher for old items.

## Discussion

In unconscious processing research, adoption of post hoc data selection has been widespread. Recent literature has underlined methodological shortcomings linked with the *post hoc subgroup selection* approach, a type of post hoc data selection which concerns selection of a subgroup of participants or trials based on a certain awareness threshold, drawing attention to the risk of misinterpreting RttM artefacts as evidence of unconscious processing (Rothkirch et al., 2022; Shanks, 2017; Shanks et al., 2021). Building upon this body of work, this article demonstrates that another pervasive form of post hoc data selection – namely, the miss-CR contrast approach – is susceptible to artifacts due to Kelley’s Paradox, a phenomenon closely associated with RttM. Recognizing the influence of Kelley’s Paradox not only offers a more parsimonious account for the findings of studies employing this approach, but also poses important theoretical implications, possibly obviating the need to postulate unconscious processes altogether.

As demonstrated through simulations, when participants from two distinct groups are selected based on their observed scores from a specified interval, a marked difference in true score means emerges between the two groups, assuming these groups are underpinned by two distributions of different true score population means. While errors are, in principle, randomly distributed, this randomness no longer holds once the selection is done based on the observed scores. Independence between error and true score does not entail independence between error and observed score; on the contrary, error positively correlates with observed score as per Eq. (1). This interplay between error and observed score causes Kelley’s Paradox, leading to a systematic misrepresentation of group differences. This is shown in our simulations, where the observed scores of advantaged participants tend to be artifactually affected by greater negative errors compared to their disadvantaged counterparts, hence concealing the difference in the true score means between the groups. This dynamic becomes especially pronounced as the error *SD* increases (i.e., as the reliability of the measure decreases), further obfuscating the difference in true score means between the groups. Taken together, the simulated results underscore the susceptibility of post hoc data selection to such statistical artifacts due to Kelley’s Paradox.

As explained, our simulations shed light on how the miss-CR contrast approach is susceptible to Kelley’s Paradox. When items are selected based on observed recognition memory, it is similar to selecting participants based on observed test scores from an interval below a certain threshold. If the recognition memory strength for an item falls below this threshold, the item is recognized as new. However, due to measurement errors, an item’s observed memory strength can differ from its true memory strength. Assuming the true memory strength mean for old items exceeds that for new ones, the true memory strength of selected old items will likely surpass that of new items. This scenario exemplifies Kelley’s Paradox, contradicting the common interpretation that these items are of equivalent memory strength simply because they have been judged as new.

In our formal analysis based on SDT, we generalized our simulation findings to contexts with unequal variances in true memory strength distributions for old and new items. Utilizing an equation derived by Arnold et al. (1993), our demonstration indicated a greater difference in expected true mean memory strengths between misses and correct rejections as the error term *SD* increases. Crucially, the equilibrium of true memory strength between misses and correct rejections – which is essential for validating unconscious memory claims – is observed only at singular points under conditions of unequal variance, and specifically when lower true mean values were assigned to true memory strength distributions for old items (i.e., at 0.5 and 1). The rarity of such equilibria suggests that the use of the miss-CR contrast approach to infer unconscious memory could only be deemed valid under extremely restricted contexts.

The formal analysis also revealed a second factor at play. The evidence for this factor, which we have termed strength skewness, becomes apparent when error is assumed to be zero. If RttM were the only factor then under these conditions misses and CRs should have equal strength, but Fig. 3 shows that this is not the case. Indeed when error is zero, misses can be stronger, equal to, or weaker than CRs, implying that even standard SDT does not predict equal strengths for misses and CRs (see Berry & Shanks, 2024, for further discussion). What is the explanation for this surprising finding, which has evidently not been appreciated by researchers employing the miss-CR contrast approach? We attribute it to an intrinsic property of normally distributed strengths, namely that these strength values will be skewed to a greater or lesser extent within any interval on the strength dimension (as explained in detail in Appendix C). In equal-variance SDT the old item distribution is more right-skewed, meaning that more of its mass is to the right, compared to the new item distribution, and this holds in all intervals. Thus, the strength of misses is always greater than that of CRs. In unequal-variance SDT, in contrast, the old item distribution can be more or less right-skewed than the new item distribution (or under very specific parameter values they could be equal). This means that the strength of misses will sometimes be greater and sometimes less than that of CRs. But regardless of the conditions, the fundamental assumption on which the miss-CR contrast approach rests is invalid.

Through a narrative review, we demonstrate the prevalent application of the miss-CR contrast approach across multiple disciplines, methodologies, and research questions. This approach is exemplified in studies ranging from neuroimaging analyses to behavioral assessments. Central to this approach is the classification of items as misses or CRs and the subsequent contrasting based on other performance metrics or attributes. Despite differences in operationalization, a recurring theme emerges: the memory strength of both item categories is considered to be equivalent, and any difference in other metrics is taken as evidence for unconscious memory. Yet, many researchers have overlooked the implications of Kelley’s Paradox, or the broader RttM phenomenon. While not dismissing all findings based on the miss-CR contrast approach, it is pivotal to acknowledge that when this method stands as the primary analytical technique, the findings risk being caused by statistical artifacts due to Kelley’s Paradox and/or strength skewness. Indeed, the susceptibility of the miss-CR contrast approach to Kelley’s Paradox is further illustrated in our experiment: during the 2AFC task, focusing on word pairs rated 1 (i.e., judged as “sure new”) from the preceding recognition stage (i.e., pairs comprising high confidence misses and correct rejections), participants consistently exhibited above-chance accuracy in correctly choosing the old words. This contradicts the intuitive presumption that old and new words rated as 1 share equivalent memory strength.

Are there any conditions in which the miss-CR contrast approach could support valid inferences about unconscious processes? One scenario that offers compelling evidence comprises an inequality in strengths for misses and CRs that is in the opposite direction to their mappings onto an implicit measure. For instance, suppose that our SDT analysis yields the necessary parameters for calculating the mean strengths of misses and CRs (*d'*, *C* for each response category, and an estimate of reliability) under an unequal-variance model and we conclude that the mean strength of misses is lower than that of CRs for high-confidence “new” responses. At the same time, we observe a behavioral measure, such as RTs in a repetition priming task, which implies greater priming for misses than CRs. These two opposing patterns (CRs stronger than misses in recognition memory, misses stronger than CRs in priming) are incompatible with any model which seeks to explain both awareness (i.e., recognition familiarity) and behavior via a single latent construct and hence would provide strong support for the involvement of an unconscious process.

But note that this inference would raise an additional set of issues if a neural measure is substituted for the behavioral one, because we would need validation of the direction of correlation between the neural signal and the underlying strength variable. Repetition suppression (Lee et al., 2020) shows that this association can often be negative. For example, the opposing and quite plausible pattern in which misses are stronger than CRs in recognition memory while a neural signal is stronger for CRs than misses would not represent strong evidence for an unconscious process. The reason is that strength and neural activation are inversely correlated in repetition suppression, so a model with a single latent factor would be sufficient to accommodate the results.

Another pattern that could provide support for unconscious processes is one in which the magnitude of the miss-CR difference is equivalent to that of the overall old-new difference (i.e., *d'* calculated across all old and new items). The RttM/skewness account always predicts that the miss-CR difference will be smaller in magnitude than the old-new difference. This can be seen for the equal-variance case in Fig. 4. In most conditions the miss-CR difference is much smaller than *d'*, although they converge under extremely conservative response criteria. Hence a pattern in which these differences are similar (and the criterion is not extreme) would be a challenge for the account.

A potential limitation to the inferences drawn here should be mentioned. The significance of strength skewness depends on the suitability of the SDT framework as a model for the data-generation process. In particular, our strength skewness analyses (Fig. 3 and 4) depend on the strengths of old and new (or target and non-target) items being normally distributed. There would be no skewness effect if, for example, the distributions were uniform (see Berry & Shanks, 2024, for further discussion). The interpretation could change even more radically if SDT was replaced by a different decision model. High-threshold theory, for example, is a discrete state model of recognition in which old items are detected as “old” with probability *d*_o_ and misidentified as new with probability 1-* d*_o_. In this account, old and new items in the non-detect (“new”) state are theoretically indistinguishable, and hence misses and CRs are truly in equivalent states of unawareness. Berry and Shanks (2024) reported fits of one particular version of high-threshold theory to experiments similar to the one reported here, in which participants were able to discriminate misses and CRs in a forced-choice test, and found that the model was able to reproduce key aspects of the results. Thus, it is possible that a high-threshold theory analysis could justify employing the miss-CR approach to identify unconscious processes. This certainly merits further exploration.

In summary, the current article demonstrates the pitfalls of the miss-CR contrast approach, particularly its susceptibility to statistical artifacts stemming from Kelley’s Paradox and strength skewness. Given the prevalence of this approach in unconscious processing research – spanning 25 years (e.g., Rugg et al., 1998) – the implications are profound. While we do not refute the possibility that previous findings derived from this approach may truly evidence unconscious processing, we submit that Kelley’s Paradox and strength skewness offer a more parsimonious interpretation. It is imperative for researchers to critically assess purported evidence of unconscious memory, ensuring that they have adequately addressed the potential confounds introduced by these two factors.

## Figures and Tables

**Fig. 7:**
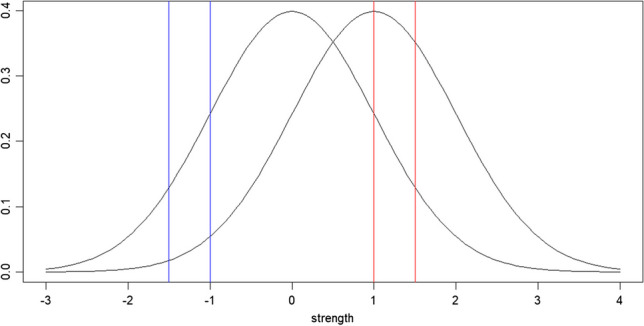
Equal variance signal detection theory. The mean of the new item distribution is 0 and that of the old item distribution is 1.0. For both distributions the standard deviation *σ* = 1.0. The blue lines demarcate the interval [–1.5, –1.0] and the red lines the interval [1.0, 1.5]

**Fig. 8:**
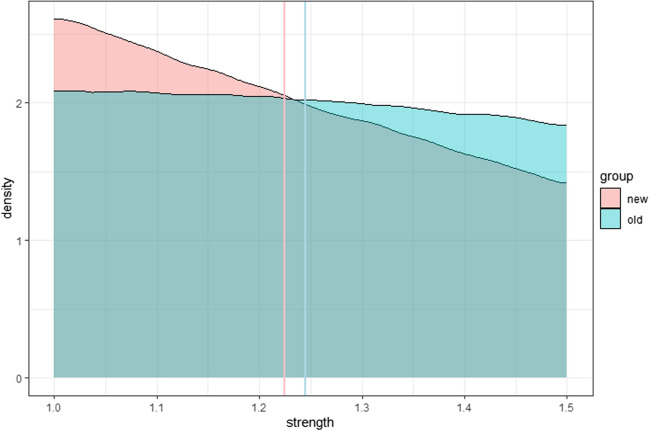
Distributions of random numbers generated from truncated normal distributions in the interval [1.0, 1.5]. The pink distribution represents new items (mean = 0, *σ* = 1.0) and the blue distribution is for old items (mean = 1, *σ* = 1.0). The vertical lines mark the resulting mean values and illustrate the key strength skewness effect

**Fig. 9:**
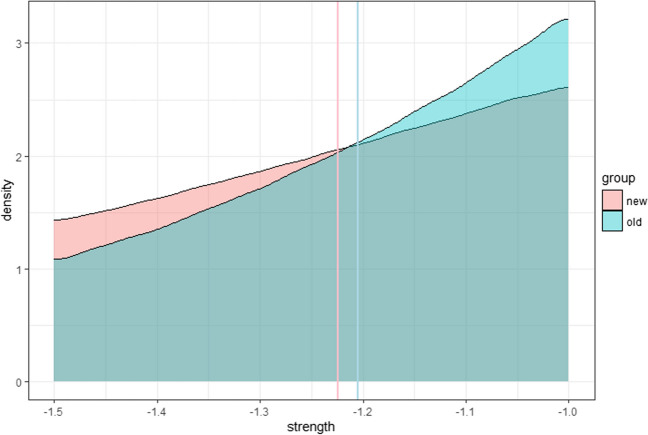
Similar to Fig. 8 but for the interval [–1.5, –1.0]. The vertical lines mark the resulting mean values and illustrate the key strength skewness effect

## Data Availability

Data from the reported experiment are openly available via the Open Science Framework at https://osf.io/t839a/.

## Appendix A

Observed scores (*X*) are created by adding error (*E*) to a true score (*T*), *X* = *T* + *E*, where *T*~*N*(*μ*_*t*_, *σ*_*t*_), *E*~*N*(0, *σ*_*e*_), and *r*(*T*, *E*) = 0. *X* is therefore distributed as $X\sim N\left({\mu}_t,\kern0.5em {\left({\sigma}_t^2+{\sigma}_e^2\right)}^{1/2}\right)$. To represent the greater scores of Group 2 compared to Group 1, *μ*_*t*_ is assumed to differ between groups. Thus, for Group 1, *μ*_*t*_ = *μ*_*t*1_, and for Group 2, *μ*_*t*_ = *μ*_*t*2_, where *μ*_*t*2_ > *μ*_*t*1_.

To obtain the subset of *X* falling within a particular range, the scores are truncated according to an upper bound *C*_*u*_ and a lower bound *C*_*l*_, where *C*_*u*_ > *C*_*l*_. To obtain the expected value of *T* in the subset of *X*, we made use of the fact that the joint distribution of *T* and *X* is a bivariate normal, with mean vector (*μ*_*t*_,  *μ*_*t*_), variance vector $\left({\sigma}_t^2,\kern0.5em {\sigma}_t^2+{\sigma}_e^2\right)$, and correlation (*ρ*), where

$$\rho =\frac{\sigma_t^2}{\sqrt{\sigma_t^2\left({\sigma}_t^2+{\sigma}_e^2\right)}}$$

When the distribution of *X* is truncated according to *C*_*l*_ and *C*_*u*_, *T* in the subset is distributed as a nontruncated marginal of a truncated bivariate normal (Arnold et al., 1993), which has the following expected value:

$$E(T)={\mu}_t+\left(\frac{-\rho }{c}\right)\left[\phi \left(\beta \right)-\phi \left(\alpha \right)\right]{\sigma}_t$$

where *ϕ* is the normal density function, $\alpha =\left({C}_l-{\mu}_t\right)/{\left({\sigma}_t^2+{\sigma}_e^2\right)}^{1/2}$, $\beta =\left({C}_u-{\mu}_t\right)/{\left({\sigma}_t^2+{\sigma}_e^2\right)}^{1/2}$, *c* = Φ(*β*) − Φ(*α*), and Φ is the cumulative normal distribution function. The expected *T* in the subset of *X* can therefore be obtained for Group 1 when *μ*_*t*_ = *μ*_*t*1_, and for Group 2 when *μ*_*t*_ = *μ*_*t*2_. *T* for Group 1 represents correct rejections while *T* for Group 2 represents misses.

The subset of *X* has a truncated normal distribution, with expected value:

$$E(X)={\mu}_t-\frac{\phi \left(\beta \right)-\phi \left(\alpha \right)}{\Phi \left(\beta \right)-\Phi \left(\alpha \right)}{\left({\sigma}_t^2+{\sigma}_e^2\right)}^{1/2}$$

## Appendix B

If *X* is a normally distributed variable with mean *μ* and standard deviation *σ*, then the equation for the expected value of a one-sided truncated normal distribution (upper tail) is:

$$E\left(X|\ X<b\right)=\mu -\sigma \frac{\phi \left(\frac{b-\mu }{\sigma}\right)}{\Phi \left(\frac{b-\mu }{\sigma}\right)}$$

where *ϕ* is the normal density function, Φ is the cumulative normal distribution function, and *b* is the truncation point (Forbes et al., 2011).

In the equal variance SDT model, the mean strength of new items, *μ*(new), can be fixed to zero without loss of generality, and *σ*(new) and *σ*(old) can similarly be fixed to 1. The mean of old items, *μ*(old), is therefore equal to ${d}^{\prime}$, and the truncation point *b* is the response criterion *C*. The expected strength of misses can therefore be written as:

$$E\left( strength|miss\right)={d}^{\prime }-\frac{\phi \left(C-{d}^{\prime}\right)}{\Phi \left(C-{d}^{\prime}\right)}$$

That of correct rejections is:

$$E\left( strength|correct\ rejection\right)=-\frac{\phi (C)}{\Phi (C)}$$

It may be possible to prove the inequality below formally

$${d}^{\prime }-\frac{\phi \left(C-{d}^{\prime}\right)}{\Phi \left(C-{d}^{\prime}\right)}>-\frac{\phi (C)}{\Phi (C)}$$

but, at least by simulation, the difference of *E*(strength | miss) – *E*(strength | correct rejection) is always positive, as shown in Fig. 4.

## Appendix C

Figure 3 (left panels) and Fig. 4 show that under the equal-variance SDT model the expected value of an interval from -∞ to a criterion *C*, *E*(*X* | *X* < *C*), is always greater under the old (i.e., misses) than the new item distribution (i.e., CRs). This makes intuitive sense as the left tail is longer under the new item distribution. For instance, if the criterion is –1.5 then the mean value for misses (based on the formula in Appendix B) is –1.82 while the mean for CRs is –1.94. The effect is caused by different degrees of skewness: skewness is –1.61 for misses and –1.44 for CRs. These are extreme degrees of skewness, with the value being more negative for misses.

Somewhat less intuitively, both the inequalities in expected value (old items > new items) and in skewness (new items > old items) hold across *all* intervals on the strength dimension.^6^ To illustrate this, we used the function rtruncnorm from the truncnorm package in R to generate 10^6^ random numbers from a truncated normal distribution with mean = 0 and *σ* = 1.0 and the same number from a truncated normal distribution with mean = 1 and *σ* = 1.0, in both cases from the interval [1.0, 1.5] illustrated by the red lines in Fig. 7. The distributions of these numbers, and their resulting means, are shown in Fig. 8. The mean is higher for old than new items (strength skewness) while skewness is lower for old (0.04) than new items (0.22): That is, the old item distribution is more symmetrical.

This relatively greater positive skew for new items reflects the fact that more of the distribution is shifted leftwards, creating a longer rightwards tail (although this has been truncated). The leftwards shifting of the distribution pulls down the mean.

Figure 9 shows the distributions for a different interval, from –1.5 to –1.0. Once again, skewness is lower for old (–0.39) than new items (–0.22) while the sample mean is greater for old items. In this case the old item distribution is more asymmetrical than the new item distribution. In analogy with Fig. 8, the relatively greater negative skew for old items reflects a longer leftwards tail, and the rightwards shifting of the distribution pulls the mean upwards.

Intervals under a normal curve with mean strength = 0 have extreme negative skewness for extreme negative strength values, gradually increasing (less negative) skewness as strength increases, have skewness = 0 when strength = 0, and have gradually larger positive skewness as strength increases further. Because the old item distribution is simply the new item distribution shifted upwards, this means that skewness is always lower under the old than the new item distribution. The old item distribution therefore always has relatively more of its mass shifted upwards within the interval compared to the new item distribution, and hence has a higher mean.

In both Fig. 8 and Fig. 9 there are more higher values for old than new items and more lower values for new than old items. For the interval [1.0, 1.5] this seems reasonable as the downward slope of the normal curve is steeper in this interval for new than for old items, as can be seen in Fig. 7. However, the relative slopes in Fig. 9 seem puzzling at first glance because, as Fig. 7 shows, the new item distribution slopes more steeply upwards in this interval than the old item distribution. How can the old item distribution simultaneously curve less (Fig. 7) and more (Fig. 9) steeply upwards than the new item distribution?

The answer is that in this interval, the likelihoods under the old item distribution are much lower than those under the new item distribution, yet in the simulation shown in Fig. 9 (and similarly for Fig. 8) we generated equal numbers of observations from each distribution. Stated differently, the blue distribution in Fig. 9 would be much lower and less steep if we had sampled in accordance with the likelihoods. However, sampling from the underlying distributions in this way would have masked the critical feature that these simulations reveal: At every point on the strength dimension, the slope of the old item distribution is steeper than that of the new item distribution if the likelihoods are equated, and hence more of its mass is towards the right.

This is easy to see analytically. For a standard normal distribution,

$$\varphi (x)=\frac{1}{\sqrt{2\pi }}{e}^{-{x}^2/2}$$

and the first derivative (i.e., slope) is

$${\varphi}^{\prime }(x)=-x.\varphi (x)$$

Hence the slope, relative to the likelihood, is simply

$$\frac{\varphi^{\prime }(x)}{\varphi (x)}=-x$$

Thus, the relative slope decreases linearly with *x*. Because the old item distribution is identical to the new item distribution but shifted upwards, the slope of the old item distribution is always greater than that of the new item distribution.

In sum, for all intervals across the strength dimension, the expected value *E*(*X*) is greater for old than for new items (strength skewness) in the equal-variance SDT model, provided ${d}^{\prime}$ > 0. At the same time, skewness itself is always greater for new than for old items. It is this skewness difference that explains the difference in mean strengths.

## Footnotes

1 The formal derivation we describe in the next section reveals that the values reported here are very close to their expected values, 44.97 and 55.03, respectively, when the selected range is 48–52, and 30.84 and 42.46, respectively, when the range is -∞–30.

2 The expected true scores are estimated based on participants with observed scores between 49.9 and 50.1. There are 330 simulated participants within this interval. These are in line with the expected values based on the formal derivation discussed below: 58.00 and 42.00 for the advantaged and the disadvantaged groups, respectively.

3 It should be noted that adding the error term to the true memory strength distributions is equivalent to adding the error term to the decision criterion.

4 The model specification was: Accuracy ∼ Rating (i.e., ratings 1-4) + (1 + Rating | *Participant*). The random effect is italicized. We included both random intercepts and random slopes in the model, in line with the recommendation by Barr et al. (2013). The 95% CI for the accuracy estimate was computed via the ‘emmeans’ package (Lenth, 2024).

5 This correlation is attenuated by the low reliability of the accuracy measure for these rating 1 trials (split-half reliability *r* = .34, 95% CI [-.43, .69], with Spearman-Brown correction; Brown, 1910; Spearman, 1910). The disattenuated correlation (Schmidt & Hunter, 2015) is *r* = -.31, 95% CI [-.88, .35].

## Code availability

R scripts for the models reported in Sections “2AFC task” and Appendix C are openly available via the Open Science Framework at https://osf.io/t839a/.
